# Immune mechanisms of idiosyncratic drug-induced liver injury

**Published:** 2017-02-12

**Authors:** Alastair Mak, Jack Uetrecht

**Affiliations:** Department of Pharmaceutical Sciences, Faculty of Pharmacy, University of Toronto, Toronto, Ontario, Canada

**Keywords:** idiosyncratic drug reactions, idiosyncratic drug-induced liver injury, immune mediated, immune tolerance

## Abstract

Idiosyncratic drug reactions (IDRs) continue to be an important issue. Specifically, idiosyncratic drug-induced liver injury (IDILI) is the most likely IDR to lead to drug withdrawal, and it accounts for a significant portion of all cases of acute liver failure. In addition, IDRs are unpredictable and their mechanisms are not well understood. There is increasing clinical evidence that most IDILI is immune mediated. Several immune mediated mechanistic hypotheses exist such as the hapten and danger hypothesis; however, they do not completely explain the idiosyncratic nature of these reactions. Extensive mechanistic studies are needed to better understand these reactions; however, it is impossible to do controlled experiments in humans, and previous animal models did not properly model IDILI. If IDILI is immune mediated and the major factor preventing liver injury in patients is immune tolerance, then a plausible method to develop an animal model of IDILI would be to impair immune tolerance. This hypothesis has shown promise in developing valid animal models of IDILI as demonstrated by a halothane induced liver injury mouse model developed by depleting myeloid derived suppressor cells (MDSCs), as well as an amodiaquine-, isoniazid-and nevirapine-induced liver injury mouse model developed by impairing immune tolerance by blocking PD-1 and CTLA-4, two immune checkpoint inhibitors. Further characterization and validation of these models is required; however, it is likely that they will make it possible to perform mechanistic studies that have been impossible in the past.

**Relevance for patients**: Idiosyncratic drug-induced liver injury can be serious leading to liver transplantation or death. Their idiosyncratic nature makes mechanistic studies very difficult. However, with the development of the first animal model that is similar to the liver injury that occurs in humans, it will be possible to study the mechanisms involved. With a better mechanistic understanding it should be possible to test drug candidates and produce safer drugs. In addition, it should be possible to design better treatments when drug-induced liver injury does occur.

## 1. Introduction

Idiosyncratic is defined as peculiar to an individual and describes idiosyncratic drug reactions (IDRs) as reactions that only affect specific individuals. In most cases, whether pharmacology, genetics, environment, or all three determine who will develop an IDR is currently not well understood. IDRs pose a significant issue for healthcare and drug development, as these reactions are often not detected in clinical trials [1]. The incidence of these reactions may only be clear after millions of people have taken the drug, where only a small percentage of patients will experience a reaction (generally <1%). In 2004 in the United Kingdom, adverse drug reactions accounted for 6.5% of hospital admissions with an overall mortality of 2% [2]. Although the percentage of IDRs is only about 10% of the total adverse drug reactions [3], given the total number of drugs prescribed, IDRs are common and represent a major and increasing cause of candidate failure in drug development. Over 10% of drugs approved during 1975-1999 acquired a black box warning or were withdrawn [4]. IDRs are especially difficult to deal with because the mechanism of injury is not well understood, and current testing is not effective in predicting their risk [1].

IDRs are described as type B adverse drug reactions, which means that they do not generally involve the pharmacological effect of the drug and do not occur in most patients. Additionally, most IDRs appear to involve the bioactivation of the drug into a reactive metabolite [5]. This is in contrast to type A reactions, which are generally a consequence of a drug’s pharmacological effect, and therefore more predictable. An example of a type A reaction is excess bleeding caused by warfarin, which is an anticoagulant. Although the incidence of serious IDRs is low, their unpredictable nature make them scary. Also, because the mechanism of these adverse reactions is poorly understood, there is no specific treatment other than withdrawal of the offending drug and supportive care [6]. While IDRs can affect a multitude of sites in the body, the three most common targets are the liver, skin, and blood cells.

Idiosyncratic drug-induced liver injury (IDILI) is the type of IDR most likely to lead to drug withdrawals [7]. In addition, IDILI accounts for 13% of all cases of acute liver failure in the USA [8]. Therefore significant research has been conducted to better understand these reactions in order to prevent future drug candidates from causing IDILI [9]. Unfortunately, as with other IDRs, the mechanism of IDILI is still poorly understood. The liver is a multifaceted organ involved in metabolism, catabolism, and digestion. In terms of metabolism, anything that is absorbed by the intestines must travel through the portal vein to the liver, where it is subject to metabolism before it enters the general circulation. Specifically, the liver plays a major role in decomposition of endogenous products and is the primary site of drug metabolism in the body. The liver is a prime target for IDRs because drug metabolism can result in the formation of reactive metabolites, and injury is most likely to occur where reactive metabolites are formed. The two major types of IDILI are hepatocellular necrosis and cholestatic liver injury. Although a specific drug usually produces a characteristic pattern of injury, it can vary in different patients.

## 2. Evidence that IDILI is immune mediated

### 2.1. Delayed onset of liver injury

Multiple clinical characteristics suggest that most IDILI is immune mediated. One important characteristic is that the onset of these reactions is generally delayed. A characteristic of adaptive immune responses is a delay in onset on first exposure to a new antigen, presumably because it takes time to expand the T cell and/or B cell population specific for the antigen. Immune responses involving adaptive immunity are pre-ceded by innate immune response and only fully come to fruition when proper antigen presentation is in place [10]. IDILI caused by many drugs appears to involve adaptive immune cells, although their role in the injury is not clearly established. IDILI liver histology is characterized by an infiltration of CD8 T cells and macrophages, with low levels of mature B cells and NK cells, and sometimes with eosinophils [11]. However it is difficult to determine if these infiltrating cells are responsible for the injury or simply a result of liver inflammation. The time to onset of IDILI varies with the drug and the patient, but most drugs that cause IDILI have a typical delay of 1-2 months [5].

#### 2.1.1. Rapid onset on re-challenge

Another important characteristic of IDILI to suggest that it is immune mediated is the rapid onset of a reaction on re-challenge with the same drug. Compared to the initial onset of IDILI, which is most often delayed, many drugs that cause IDILI will cause a more immediate reaction when a patient is given the same drug again [12,13]. This characteristic supports the hypothesis that IDILI is immune mediated because the most plausible mechanism for this more rapid onset is immune memory. Memory immune cells have already been primed to the specific antigen and do not require the delay that naïve immune cells need in order to be primed. Additionally, re-challenge often results in a more severe reaction because memory immune cells are able to mount a stronger immune response than during the initial reaction [13]. Although rapid onset of a reaction on re-challenge is common to many drugs that cause IDILI, occasionally a second exposure will not generate any reaction or the second reaction will be delayed similarly to the first reaction [5,14]. This is especially true if the initial reaction was mild. Ximelagatran-induced liver injury or isoniazid (INH)-induced liver injury does not usually recur on rechallenge, and in the case of INH, many patients can be successfully restarted on the drug, especially if there is a slow dose escalation to the therapeutic dose [15,16]. Therefore it is possible that a protective adaptation in immune response can develop in some individuals.

#### 2.1.2. Positive lymphocyte transformation tests

Positive lymphocyte transformation tests (LTTs) also suggest that IDILI is immune mediated. A LTT involves measuring the proliferation of lymphocytes (isolated from a patient with IDILI) when exposed to a drug in vitro [17]. This indicates that the lymphocytes isolated from an IDILI patient have been sensitized to the drug that caused the liver injury [17]. In the case of INH, patients who develop mild INH-induced IDILI only had a positive LTT when the lymphocytes are exposed to INH-modified proteins. While patients who develop severe INH-induced IDILI had a positive LTT to INH-modified proteins and INH itself [17]. This suggests that it was drug-modified proteins that initiated the immune response, but there was epitope spreading with a strong immune response leading to more severe injury. The LTT is a useful test for diagnosis of IDILI, although the false negative rate for INH IDILI is about 50%, and it varies depending on the drug and the IDR [18,19].

#### 2.1.3. Increase in inflammatory markers

IDILI is also sometimes associated with the formation of anti-drug antibodies, and also an increase in pro-inflammatory cells and cytokines. Antibodies are an important part of adaptive immunity and aid in the recognition of antigens. Although anti-drug antibodies are sometimes found in the serum of individuals who have developed IDILI, it is not known if these antibodies are pathogenic or not [20]. Also, similar to the LTT in INH-induced liver injury, anti-drug antibodies were only found in severe INH-induced IDILI [21]. Mild INH-induced IDILI has also been reported to involve pro-inflammatory Th17 cells and T cells producing IL-10 [21]. The testing for anti-drug antibodies is limited by the lack of availability of suitable antigens that can be used for such testing.

#### 2.1.4. HLA associations

Recently there have been several HLA associations found relating to IDRs. The human leukocyte antigens (HLA) are genes that most notably encode cell-surface antigen-presenting proteins. Genetic associations can be useful in order to screen patients who would be at an increased risk of an IDR. However, in most cases, even if a patient is treated with the drug associated with the incriminated HLA, they are unlikely to have an IDR. An exception is the association between HLA*B5701 and abacavir-induced hypersensitivity reactions, which can also effect the liver. The incidence of hypersensitivity reactions in patients who carry the HLA*B5701 gene is greater than 50%, and this adverse reaction was abolished through HLA-B*5701 screening [22]. In studies conducted in North America, Europe, and Australia, the HLA-B*5701 test sensitivity was 46-78% [23]. Additional HLA associations for IDILI include flucloxacillin, which is associated with HLA-B*5701, ximelagatran, which is associated with HLA-DRB1*07:01, amoxicillin/clavulanic acid, which is associated with HLA-DRB1*15:01, and INH, which is associated with HLA-DQB1* 02:01 [24-27]. However the strength of these HLA associations varies depending on the drug and are generally weak.

#### 2.1.5. Immune tolerance

**Figure 1.**
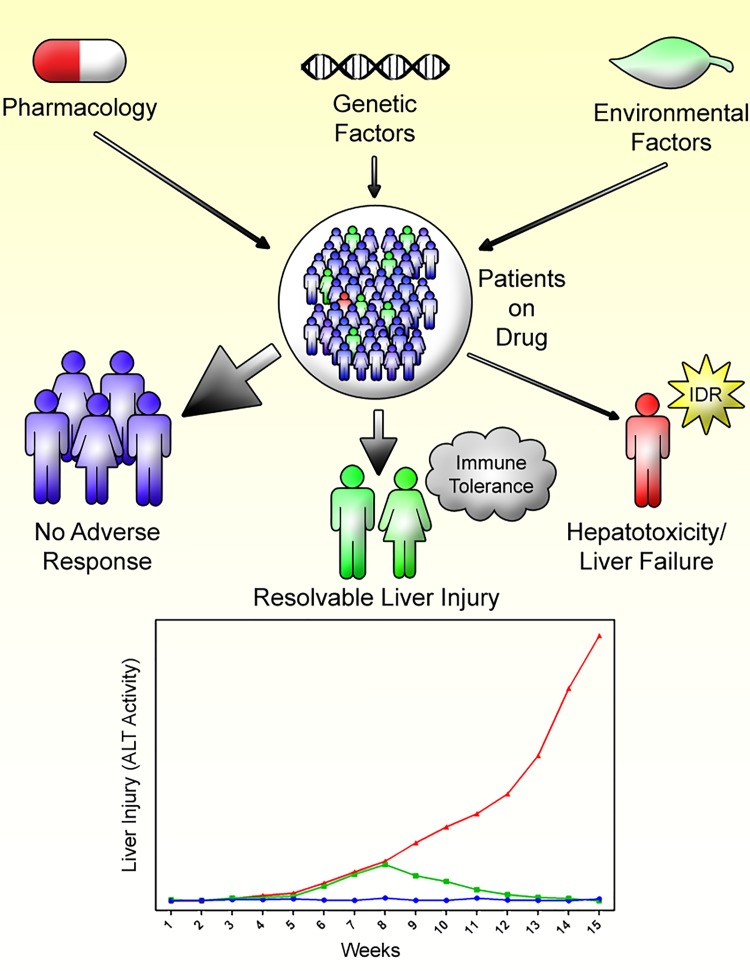
Pharmacology, genetics, and the environment may all play roles in determining who will develop an IDR, in this case liver injury. In general there is a delay in the onset of injury, and depending on the individual, a patient may have no clinically evident liver injury, develop mild liver injury that resolves despite continued treatment, or develop liver failure. The characteristics such as time to onset are similar in the mild and serious injury

Although the aforementioned characteristics favor the immune system causing IDILI, this is balanced by immune tolerance. This usually results in the elimination of foreign antigens without resulting in unnecessary tissue damage. Immune tolerance is an important characteristic that is likely the ultimate response in most patients to drugs that can cause IDILI. For a drug known to cause IDILI, most patients will experience no apparent injury [5]. Whether this is due to pharmacological, genetic, or environmental factors is not well understood, and these factors may vary with different drugs and in different individuals. A significant percentage of patients will, however, develop mild liver injury that resolves despite continued treatment. Up to 20% of INH-treated patients will develop a small increase in ALT that returns to normal despite continued treatment [28]. These patients may have the specific pharmacological, genetic, or environmental factors necessary to develop mild injury; however, further injury is subdued by immune tolerance. Finally, an even smaller percentage of total patients will develop severe liver injury, and this may involve the aforementioned factors as well as the failure to develop immune tolerance, and therefore the inability to adapt to the mild injury (Figure 1).

## 3. Mechanistic hypotheses

There are two major hypotheses for the mechanism of immune mediated drug-induced liver injury; specifically, the Hapten and Danger hypotheses. These two hypotheses are complementary in that both formation of a hapten and the production of danger signals may be required to induce an immune response. Additional hypotheses that help to explain the characteristics of IDRs have also been proposed, which include molecular mimicry, heterologous immunity, and inflammasome activation. Non-immune hypotheses such as mitochondrial injury and bile salt exporter pump inhibition have also been proposed. Even if IDILI is immune mediated, these mechanisms may contribute to inducing an immune response.

### 3.1. Hapten hypothesis

**Figure 2.**
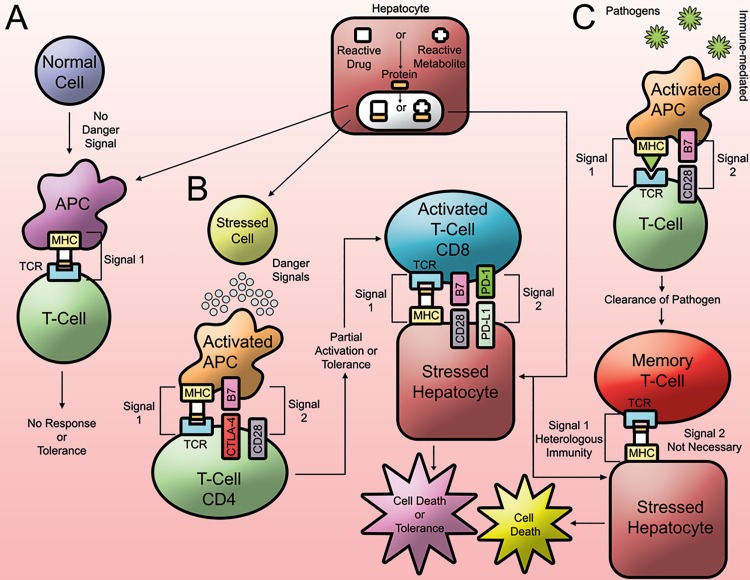
Hypotheses of immune mediated IDRs. A) The Hapten hypothesis: a reactive drug or reactive metabolite acting as a hapten binds to endogenous proteins, creating drug-modified proteins and generating Signal 1 of the immune response. B) The Danger hypothesis: reactive species damage cells, resulting in the release of danger signals and leading to Signal 2 of the immune response. Immune cells such as CD8 T cells that have received Signal 1 and 2 can then cause cell death. C) Molecular mimicry and heterologous immunity: previous exposure to pathogens can prime the immune system, create memory immune cells that recognize subsequent drug-modified proteins, and lead to a strong immune response and cell death.

The Hapten hypothesis involves a reactive drug or a reactive metabolite of a drug acting as a “hapten” and binding to endogenous proteins. In the case of IDILI, as the primary site of drug metabolism, many reactive metabolites can be formed and then bound to liver proteins. This drug-modified protein adduct can then be taken up by antigen presenting cells (APC) and presented to T cells on the major histocompatibility complex (MHC) to produce signal 1 of an immune response (Figure 2A). The drug-modified proteins are seen as “foreign” by the immune system, and that is what leads to an immune response. Endogenous liver proteins are recognized by the immune system as “self”, but when the proteins are bound to drugs the immune system can recognize it as “nonself”. An active immune response is activated towards “nonself”, while tolerance results from recognizing “self”. Small molecules do not elicit an immune response unless they covalently bind to proteins because in most cases small molecules do not bind with sufficient affinity to the MHC [29]. Most drugs that are associated with a significant incidence of IDRs are metabolized to reactive metabolites that could act as haptens and lead to an immune response. However, not all drugs that are metabolized to reactive metabolites are associated with a significant incidence of IDRs. Therefore the Hapten Hypothesis is likely only a part of the mechanism because it is still not clear what determines which drugs will cause IDILI [30].

Experiments performed with INH, amodiaquine (AQ), and nevirapine (NVP) treatment in mice, all drugs known to cause IDILI in humans, showed significant covalent binding to liver proteins. Anti-INH, AQ, and NVP antibodies were developed to detect drug-modified proteins, and when liver proteins were run on a western blot and stained with the antibodies, there was significant binding of these drugs to a wide range of proteins in their respective blots [31,32,33]. INH, AQ, and NVP all have the ability to be converted to a reactive metabolite. These experiments with anti-drug antibodies, therefore, demonstrate that treatment with these drugs produce drugmodified liver proteins. Although drug-modified proteins have been shown to form, how the adduct elicits an immune response is not well understood. As described in the previous experiments, there are a multitude of drug-modified proteins formed, and therefore it is difficult to determine which one is responsible for causing IDILI. Additionally, in the sera from patients with INH-induced liver failure, antibodies against CYP2E1 modified by INH were found, and INH was found to form covalent adducts with CYP2E1, CYP3A4, and CYP2C9 in vitro [21]. Although there is evidence of reactive metabolites of drugs acting as haptens to create drug-modified proteins, it is unknown what their role in the development of IDILI might be.

### 3.2. Danger hypothesis

The Danger Hypothesis complements the Hapten Hypothesis by providing signal 2 of the immune response. Signal 2 consists of costimulatory signals that originate from activated APCs and are required for activation of T cells (Figure 2B). Classic signal 2 receptors include B7 (CD80, CD86) on APCs binding to CD28 on T cells. Costimulation is required in addition to signal 1. The T cell requires signal 2 to verify if it should be activated. Without signal 2 the response is likely to be immune tolerance, and therefore no clinical immune response and no adverse reaction. Danger signals are a likely mechanism by which APCs are activated and produce signal 2. Signal 2 is important as verification so that the immune system is only activated by something that is causing injury or is dangerous to an organism. Therefore, it is possible that damage to cells can cause the release of danger signals that stimulate an immune response [34]. Common danger signals include HMGB1, DNA, RNA, and other nuclear and cytosolic proteins [35]. Characteristically, these danger signals originate from within a cell, and when they are released from a dying cell, they can be recognized by APCs and trigger their activation. Reactive metabolites of drugs have the potential to cause cell injury, and therefore cause the release of danger signals. In an experiment involving AQ treatment in rats, AQ was shown to cause direct cytotoxicity that preceded the liver injury, and HMGB1 was significantly increased in the serum 6 hours after the first dose [36]. However, the type of danger signal can vary depending on the drug that is causing the damage as well as the different types of cells being affected. The danger hypo-thesis is unable to explain the mechanism of IDILI alone; however, when combined with the hapten hypothesis, they complement each other. These two hypotheses together suggest that in order for a drug to cause IDILI, both signal 1 and signal 2 of the immune response must be present.

The danger hypothesis suggests that danger signals produced from other factors such as liver infections may increase the incidence of IDILI. Although there appears to be exceptions, in general, it does not appear that preexisting liver disease increases the risk of IDILI [37]. However, such patients have a lower liver reserve, and in a prospective study looking at IDILI patients, it was found that IDILI appeared to be more severe in patients with a preexisting liver disease than in those without. Therefore pre-existing liver disease is associated with significantly higher patient mortality [6]. This relationship does not always follow, possibly due to the variance in pre-existing liver diseases as well as the resulting danger signals. Inflammation can play an important role in generating danger signals to stimulate an adaptive immune response. This theory was used in the inflammagen model involving co-exposure of drugs such as ranitidine with lipopolysaccharide (LPS) in rats, resulting in more liver injury than with the drug alone [38]. Unfortunately, this model is different in every important respect from IDILI in humans; it more resembles LPS-induced liver injury. Poly (I:C) and CD40 agonists, both immunostimulants, have also been used to increase the liver injury caused by halothane in C57BL/6J mice; however, this did not result in a model of delayed onset liver injury similar to the liver injury caused by halothane in humans [39]. It appears that in most cases, a simple co-treatment of a drug and an immunostimulant is unable to overcome immune tolerance.

### 3.3. Molecular mimicry and heterologous immunity

If IDILI is immune mediated, it is possible that an individual’s prior exposure to antigens may affect their susceptibility to IDILI. Recently an experiment was designed to determine how previous exposure to a related antigen would affect the extent of subsequent drug induced injury. The experiment utilized immunization of mice with AQ-modified liver proteins prior to AQ treatment. However, mice immunized with AQ-modified proteins were paradoxically resistant to AQ-induced liver injury, and immunization was associated with an increase in cells associated with immune tolerance [40]. This experiment describes a scenario were the immune system is primed to an antigen related to the subsequent drug-induced liver proteins; however, either the initial immune system priming was not strong enough to overcome immune tolerance, or memory immune cells were unable to recognize the subsequent drug induced proteins. Therefore, a follow up experiment utilized anti-CTLA-4 and antiPD-1 antibodies during the immunization period to impair immune tolerance and attempt to increase immune system priming. Mice immunized with AQ-modified proteins and treated with anti-CTLA-4 and anti-PD-1 antibodies showed increased liver injury compared to mice treated with AQ alone (Mak and Uetrecht, unpublished results). However, all mice treated with AQ at some point recovered despite continued treatment. Therefore, previous exposure to antigens may increase the risk of IDILI; however immune system priming must be very strong.

Although the previously described experiment utilized a similar antigen for the immunization, the subsequent antigen does not have to be similar. An individual’s repertoire of memory immune cells is shaped by every exposure to antigens. Therefore subsequent drug-induced adverse drug reactions may involve an immune cell’s cross reactivity between a prior antigen and the resulting drug-modified proteins produced (Figure 2C). An immune response to a pathogen can therefore shape an individual’s immune system and what it can react to in the future. Cross reactivity describes an immunological occurrence where a complex antigen with different macromolecules can mount multiple immune responses to these different epitopes [41]. Therefore cross reactivity can cause an immune response to another antigen with at least one similar epitope as the previous antigen; this is referred to as molecular mimicry. Additionally, the immune system is even able to recognize varying antigen epitopes by interacting on different parts of the T cell or B cell receptor [42]. This is referred to as heterologous immunity in which an immune response to one pathogen can provide immunity to another unrelated pathogen. Therefore a strong immune response to a pathogen could overcome immune tolerance via heterologous immunity and lead to IDILI. Heterologous immunity provides an attractive hypothesis to explain the idiosyncratic nature of IDILI; however, it will be very difficult to prove, and it would be very difficult to predict in humans.

### 3.4. Inflammasome activation

**Figure 3.**
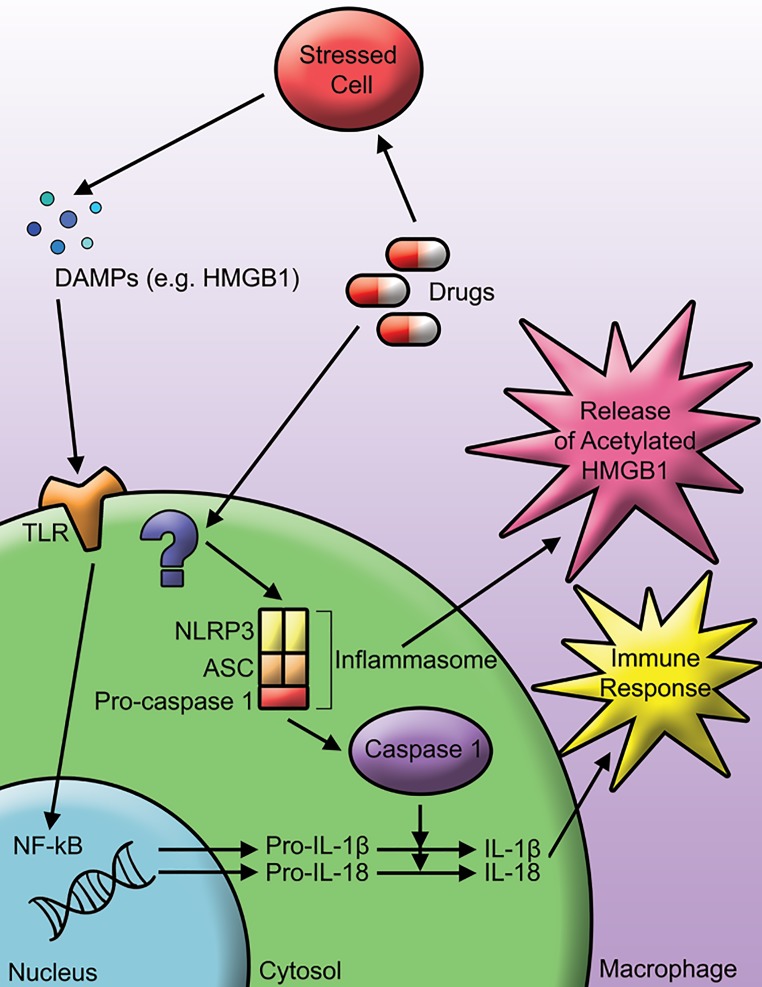
Inflammasome activation. Drugs have the potential to activate the inflammasome, leading to release of IL-1ß and IL-18, which can further stimulate an immune response.

Inflammation can be a protective immune response that is initially triggered by the innate immune system in response to harmful stimuli, dead cells, or danger signals. However, there is a balance between sufficient inflammation necessary to eliminate a persistent infection and excessive inflammation that can cause inflammatory diseases. These innate immune functions rely on the recognition of pathogen-associated molecular patterns (PAMPs), and danger-associated molecular patterns (DAMPs) by pattern-recognition receptors (PRRs) [43]. Drugs, or their reactive metabolites, have the potential to cause cell damage and subsequent release of DAMPs to initiate an inflammatory response. Following recognition of danger signals, activation of inflammasomes in innate immune cells is necessary for the innate immune system to mount an immune response. The inflammasome is a combination of innate immune receptors and sensors that regulate the activation of caspase-1 in order to induce inflammation. Inflammasomes are protein complexes that assemble in the cytosol after recognition of PAMPs or DAMPs [44]. There are many families of PRRs and therefore inflammasomes; however, well studied examples include the NOD-like receptors (NLRs) and the absent in melanoma 2 (AIM)-like receptors [45]. Therefore well-known inflammasomes include the NLRP3 inflammasome and the AIM2 inflammasome. The inflammasome acts to recruit inactive pro-caspase-1 and leads to autoproteolytic cleavage and activation of caspase-1 [46]. Active caspase-1 can cleave pro-IL-1ß and pro-IL-18 into their active pro-inflammatory forms, ready to be released from the cell to induce inflammation. Activated caspase-1 is also capable of inducing an inflammatory form of cell death known as pyroptosis [47]. Over activation of the NLRP3 inflammasome has been shown to result in shortened survival, poor growth, hepatocyte pyroptosis, severe liver inflammation, and fibrosis in mice [48]. Although inflammasomes are important in innate immunity to fight off infection, excess inflammation can lead to a variety of autoinflammatory conditions. In relation to IDILI, danger signals or drug-modified proteins may signal the activation of inflammasomes and lead to immune mediated liver injury (Figure 3).

Recently an in vitro experiment was designed to evaluate the ability of certain drugs that cause idiosyncratic skin rashes to activate inflammasomes. THP-1 cells (a human monocyte cell line) were treated with two pairs of chemically similar drugs. Telaprevir has a “black box” warning for severe skin rashes while boseprevir does not, and dimethyl fumarate causes contact sensitization and ethacrynic acid does not cause idiosyncratic reactions even though it covalently binds to proteins. Telaprevir and dimethyl fumarate activated inflammasomes while boseprevir and ethacrynic acid did not [49]. This suggests that inflammasome activation with production of IL-1ß may be a biomarker of IDR potential. Drugs that require bioactivation into their respectful reactive metabolites need to be tested in this model, as well as drugs that cause other forms of IDRs such as IDILI or blood disorders. IDILI may be difficult to study in this model because drugs that cause IDILI generally need to be converted to their reactive metabolite in order to cause liver injury. However, inflammasomes appear to be an intriguing hypothesis of how reactive metabolites activate the immune system leading to an IDR.

### 3.5. Non-immune hypotheses

There are several non-immune hypotheses for IDILI including metabolic idiosyncrasy, mitochondrial injury, endoplasmic reticulum (ER) stress, and bile salt export pump (BSEP) inhibition. Metabolic idiosyncrasy describes an individual’s genetic idiosyncrasy in biotransformation of a drug relating to the risk of developing IDILI. Although polymorphisms in biotransformation, e.g. the slow acetylation pheno-type is associated with an increased risk of INH-induced liver injury, such associations are quite weak and cannot explain the idiosyncratic nature of IDRs [50]. Mitochondrial injury, ER stress, and BSEP inhibition are all hypotheses that involve the drug or a reactive metabolite of a drug disturbing cellular homeostasis and therefore causing cell injury. Although these hypotheses, by themselves, have difficulty explaining the characteristics and idiosyncratic nature of IDILI, cell injury could produce danger signals and lead to an immune response. Overall, these non-immune hypotheses may play a role in the mechanism of IDILI; however, they may play more of a setup role for the immune system.

## 4. Animal models with impaired immune tolerance

**Figure 4.**
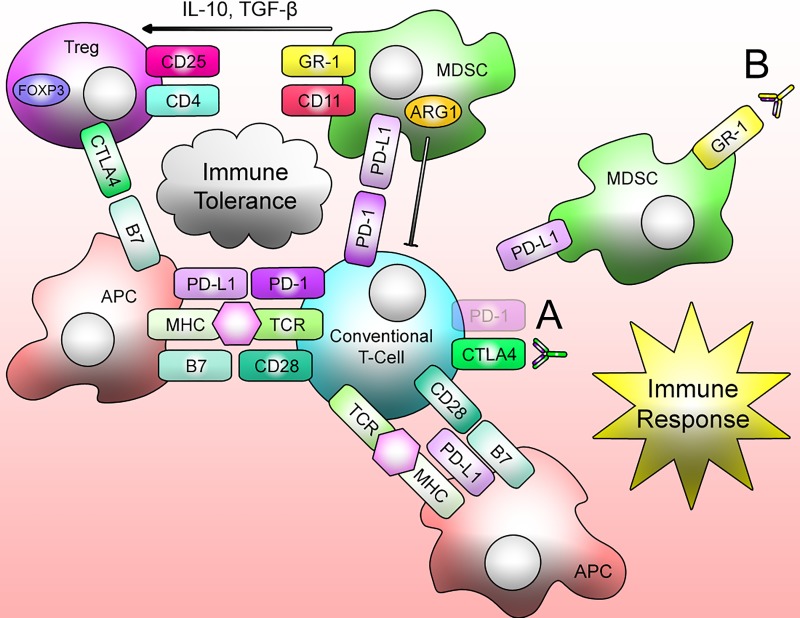
There are multiple immune tolerance pathways including CTLA-4 and PD-1 signalling from regulatory immune cells such as Treg cells or MDSCs. Therefore immune tolerance can be impaired by (A) interfering with signalling pathways by using PD-1-/-mice lacking the PD-1 receptor or using anti-CTLA-4 antibodies, or (B) depleting MDSCs using anti-Gr-1 antibodies.

If IDILI is immune mediated and most patients adapt to mild liver injury, this adaptation is likely to involve immune tolerance. Therefore a reasonable method to develop animal models of IDILI would involve immune system modulation focused on impairing immune tolerance. A recent strategy for the treatment of cancer has been to impair immune tolerance so that the immune system targets cancer cells, which often express antigens not present on normal cells. This treatment generally involves antibodies that directly target certain immune cells or receptors. Therefore the following experiments describe animal models of IDILI developed by using these antibodies to impair immune tolerance (Figure 4).

### 4.1. Depletion of myeloid-derived suppressor cells

In an experiment by Chakraborty et al. [51], a model of halothane-induced allergic hepatitis was developed in mice when myeloid-derived suppressor cells (MDSCs) were depleted. Halothane is an anesthetic agent that can cause lethal hepatotoxicity in patients [52]. Characteristically, as a drug that causes IDILI, it can cause self-limiting elevations in ALT or severe hepatotoxicity. The exact mechanism of halothane-induced IDILI is not well understood; however, there is evidence that the reaction is immune mediated [53]. The characteristic self-limiting liver injury suggests that most patients adapt to the drug, and this adaptation may involve immune tolerance. The lack of a valid animal model of halothane-induced IDILI has made it difficult to study its mechanism of injury.

In the experiment by Chakraborty et al. [51], female Balb/cJ mice were initially injected intraperitoneally with 30 mmol/kg of halothane and displayed the self-limiting ALT increases seen in humans. Leukocytes infiltrating the liver were found to be predominantly CD11b+Gr1 high cells and were further characterized as MDSCs due to their immunosuppressive abilities. MDSCs are a heterogenous population of cells that have strong immunosuppressive abilities. They typically expand during any strong inflammatory event and can suppress T-cell responses through cell-cell contact [54]. In mice, MDSCs are broadly defined as cells that express CD11b and GR1. Therefore, anti-Gr1 was used to deplete MDSCs and impair immune tolerance. The depletion of MDSCs prior to initial halothane treatment resulted in increased liver injury nine days post halothane rechallenge. This injury was characterized by an increase in IL-4, and infiltration of eosinophils, CD4+ T cells, and CD8+ T cells. Further characterization of the injury showed that depleting CD4+ T cells protected the mice from liver injury. Overall, it appears that impairing immune tolerance by depleting MDSCs is able to unlock the potential of halothane to cause liver injury. However, even in this experiment the injury ultimately resolved. Additional drugs need to be tested in this animal model to validate it as a general model of IDILI.

### 4.2. Inhibition of immune checkpoint receptors

In an experiment by Metushi et al., [32], treatment of PD-1^-/-^ mice with anti-CTLA-4 and AQ led to significant liver injury that was sustained throughout treatment. AQ is an aminoquinoline used as an anti-malarial medication. It has a history of causing severe IDILI that can be fatal, and therefore is no longer used for malaria prophylaxis [55]. The exact mechanism of AQ-induced IDILI is again not well understood, partially due to a lack of a valid animal model [56]. However, it is known that AQ is metabolized into N-desethylamodiaquine (DEAQ) by CYP2C8, and both the parent drug and this metabolite can be oxidized to a reactive quinonimine metabolite [57,58]. Characteristically, as a drug that causes IDILI, only a small proportion of patients will develop severe liver injury, and the onset of injury is usually delayed with an onset after 1-4 months [59]. In a previous experiment involving treatment of wild type C57BL/6 mice with AQ alone, mice developed mild liver injury; however, they recovered despite continued treatment [32]. This recovery was hypothesized to be due to immune tolerance; therefore, two immune checkpoints were targeted to try to impair immune tolerance.

PD-1 and CTLA-4 are negative regulators of T cell activation and are important for the induction of immune tolerance [60]. Although impairing immune tolerance in the treatment of cancer has been partially successful, there are several redundant mechanisms of immune tolerance. Therefore blocking multiple immune checkpoint pathways has shown greater promise for the treatment of cancer [61]. In particular, combination therapy concurrently targeting PD-1 and CTLA-4 immune checkpoints has shown remarkable antitumor effects [61]. The interaction between the PD-1 receptor and its ligand 1 and 2 (PD-L1/2) is a key pathway to suppress an immune response. PD-1 is expressed on T cells, B cells, monocytes, natural killer cells, and many tumor-infiltrating lymphocytes [62]. PD-L1 and -L2, when bound to PD-1, inhibit T-cell proliferation, cytokine production, and cell adhesion. Engagement of PD-1 and its receptors causes induction of PD-1 on activated T cells and thus aids in preventing autoimmunity and protection against tissue damage when the immune system is activated in response to infection [63]. CTLA-4 is expressed on T cells and binds to CD80 and CD86 to cause negative regulation of T cell-mediated immune responses. CD80 and CD86 are also ligands for CD28 on T cells and this interaction aids in costimulation leading to T cell proliferation, cytokine production, and survival [64]. Following MHC-peptide/TCR signaling, stronger TCR signals result in greater recruitment of CTLA-4 [65]. Small amounts of CTLA-4 can out-compete CD28 and attenuate T cell responses because CTLA-4 binds to CD80/CD86 with more affinity than CD28 [68]. Therefore the experiment by Metushi et al. [32], utilized PD-1^-/-^ mice that completely lacked the PD-1 protein and an anti-CTLA-4 anti-body to block the interaction of CTLA-4 with its receptor.

PD-1 and CTLA-4 are expressed on a large proportion of tumour infiltrating lymphocytes in many different cancers [67-69]. The expression of these molecules promote immune tolerance and protect the tumours from attack by the immune system. Additionally, tumour cells from many types of cancers also express high levels of the major PD-1 ligand, PD-L1 [70,71]. Aside from cancer, PD-1 and CTLA-4 expression was elevated in other immune mediated diseases such as acute hepatitis A infection [72], hepatitis C infection [73], and HIV infection [74]. This may seem ironic, but the immune system must keep a balance between an immune response that can destroy pathogens and an excessive reaction that causes tissue damage. In terms of IDRs, although most reactions are believed to be immune mediated, immune tolerance has not received much attention, and there is nothing published on the expression levels of PD-1 and CTLA-4 in these adverse reactions. In a study by Metushi et al. [21], mentioned previously, patients taking INH as a precaution with no active tuberculosis were recruited. Blood samples were taken from these patients over time and their peripheral blood mononuclear cells (PBMCs) were phenotyped for changes over time. Although it was unlikely that a patient in this experiment would develop IDILI, 6 out of 16 patients did develop a small increase in ALT during INH treatment. Although in this experiment PD-1 and CTLA-4 expression was not evaluated, the patients that developed a small increase in ALT showed a significant increase in in T cells producing IL-10. IL-10 is considered an anti-inflammatory cytokine and is involved in immune tolerance. Follow-up studies to assess PD-1 and CTLA-4 expression in the same category of patients have been attempted in our lab; however, patient recruitment levels have been low and therefore there is no complete data as of now.

In the experiment by Metushi et al. [32], PD-1^-/-^ mice were treated with 250 µg of anti-CTLA-4 IP weekly and given 0.2% w/w AQ mixed in rodent meal *ad libitum*. This treatment resulted in significantly increased ALT levels compared to controls, and the liver injury was sustained throughout treatment unlike the recovery seen in mice treated with AQ alone. This injury was characterized histologically by significant infiltration of lymphocytes and evidence of piecemeal necrosis. The histological findings are similar to what is seen in humans [11]. The liver injury was characterized by flow cytometry, and there were significant increases in infiltrating T regulatory cells and CD8+ T cells. Subsequent experiments showed that this animal model resulted in significant liver dysfunction as measured by increases in total bilirubin, and defined CD8 T cells as the likely cause of the liver injury [40]. Follow-up experiments used PD-1^-/-^ mice treated with anti-CTLA-4 and INH or NVP, both drugs known to cause IDILI in humans, to test if this animal model could be a general model for IDILI. This impaired immune tolerance animal model treated with INH or NVP developed significantly increased liver injury compared to INH or NVP alone [40]. Therefore, this animal model appears to be able to unlock the potential of multiple drugs to cause IDILI. This animal model will allow for better testing of mechanistic hypotheses and therefore better understanding of IDILI. Additionally, this animal model also has the potential to work as a screening tool for drug development; however, it is unlikely to work with every drug, especially drugs that have a strong HLA requirement.

## 5. Conclusions

There is a large amount of clinical evidence that suggests most IDILI is immune mediated. From delayed onset liver injury, rapid onset on rechallenge, HLA associations, positive lymphocyte transformation tests and elevated proinflammatory cells and cytokines. However, due to the idiosyncratic nature of these reactions and the previous lack of valid animal models, the exact mechanism of IDILI is still not well understood and mechanistic hypotheses have been difficult to test. As mentioned earlier, it is not known whether pharmacology, genetics, environment, or all three sway the specificity an IDR to an individual. There are several mechanistic hypotheses that directly involve the immune system, as well as non-immune hypotheses that may also be involved and not necessarily separate from the immune hypotheses. As mentioned earlier, the hapten hypothesis complements the danger hypothesis, because a successful immune response requires both signal 1 and signal 2 to occur. Differences in the metabolism of a drug can affect the amount of reactive metabolite formed and subsequent production of drug-modified proteins (hapten hypothesis) and amount of cell damage (danger hypothesis). However, all associations between the risk of IDILI and polymorphisms in drug-metabolizing enzymes that have been observed to date have been weak. Additionally, ER stress, mitochondrial injury and BSEP inhibition may generate danger signals leading to antigen presenting cell activation; however, the predictive value of in vitro assays to quantify these effects is controversial. Therefore, it will be difficult to determine the exact mechanism of IDILI as many of these hypotheses may be linked.

In order to better test these mechanistic hypotheses there must be good animal models or a plentiful supply of human samples. As the latter are not available, generation of valid animal models is a must to better understand this injury. Previous animal models of IDILI involved high doses of the drug, acute injury, and histology that did not resemble the injury in humans [56]. As mentioned previously, impairing immune tolerance in the form of anti-Gr1 antibodies, PD-1^-/-^ mice and anti-CTLA-4 antibodies has resulted in the first valid animal models of IDILI that include many characteristics similar to what is seen in human IDILI. Further characterization of these animal models along with tests with other drugs that cause IDILI will allow for better understanding of the mechanism of IDILI.
